# Splicing QTL of human adipose-related traits

**DOI:** 10.1038/s41598-017-18767-z

**Published:** 2018-01-10

**Authors:** Liang Ma, Peilin Jia, Zhongming Zhao

**Affiliations:** 1 Center for Precision Health, School of Biomedical Informatics, The University of Texas Health Science Center at Houston, Houston, TX USA; 2 Human Genetics Center, School of Public Health, The University of Texas Health Science Center at Houston, Houston, TX USA

## Abstract

Recently, genome-wide association studies (GWAS) have identified 11 loci associated with adipose-related traits across different populations. However, their functional roles still remain largely unknown. In this study, we aimed to explore the splicing regulation of these GWAS signals in a tissue-specific fashion. For adipose-related GWAS signals, we selected six adipose-related tissues (adipose subcutaneous, artery tibial, blood, heart left ventricle, muscle-skeletal, and thyroid) with the sample size greater than 80 for splicing quantitative trait loci (QTL) analysis using GTEx released datasets. We integrated GWAS summary statistics of nine adipose-related traits (an average of 2.6 million SNPs per GWAS), and splicing QTLs from 6 GTEx tissues with an average of 337,900 splicing QTL SNPs, and 684,859 junctions. Our filtering process generated an average of 86,549 SNPs and 162,841 exon-exon links (junctions) for each tissue. A total of seven exon-exon junctions in four genes *(AKTIP*, *DTNBP1*, *FTO* and *UBE2E1*) were found to be significantly associated with four SNPs that showed genome-wide significance with body fat distribution (rs17817288, rs7206790, rs11710420 and rs2237199). These splicing events might contribute to the causal effect on the regulation of ectopic-fat, which warrants further experimental validation.

## Introduction

Adiposity is one of the leading risk factors for type 2 diabetes^1^, hypertension and cardiovascular disease^2^. Recent studies have suggested that body fat distribution, above and beyond generalized adiposity, is an important metric of metabolic health, as specific fat compartments are associated with the corresponding metabolic risk^3^. Adiposity is highly heritable: the heritability of subcutaneous adipose tissue and visceral adipose tissue was previously estimated to be 57% and 36%, respectively^4,5^.

Recently, genome-wide association studies (GWAS) of nine traits related to body fat distribution were conducted in up to 9,594 women and 8,738 men of European, African, Hispanic and Chinese ancestries, with and without sex stratification^6^. A total of 11 loci have been identified from 27 genomic scans and 7 loci are new. To translate the new discoveries into clinical care, the exact physiological mechanisms underlying each genotype-phenotype association remains to be disentangled. Importantly, all the 11 loci are not in the protein-coding regions (Supplementary Table S1). Further analysis of their regulatory roles will be an important task to decipher their genetic susceptibility to body fat.

Alternative splicing is the process by which different splice sites in precursor messenger RNA are selected to produce multiple distinct messenger RNA isoforms. Splicing plays the lead role in shaping the post-transcriptional landscape and is the main mechanism governing protein diversity. Alternative splicing normally affects multi-exon genes often in a cell type-, condition-, tissue- or species-specific fashion^7^, and modulates phenotypic changes in a flexible and dynamic way^8^. Disease is often found to be initiated when alternative splicing undergoes dysfunction^9^. RNA-sequencing (RNA-seq) data is mainly used to study gene level expression. However, it can be used for the quantification of expression splicing variants, such as splice junctions. We refer to the quantitative measurement of isoforms and splicing junctions associated with the genetic variation as splicing QTLs. Splicing QTLs will help identify SNPs that are associated with variation in the expression levels of exon-exon junctions.

In this study, we systematically investigated the events of slicing QTLs of the significant GWAS SNPs for body fat distribution in order to gain a deep understanding of potential mechanisms of the regulatory roles. Augmented with the largest splicing QTLs from the GTEx project^10^, we identified 7 splicing events involving the SNPs that were significantly associated with 3 body fat distribution phenotypes.

## Results

### Summary statistics and integration

**Figure 1:**
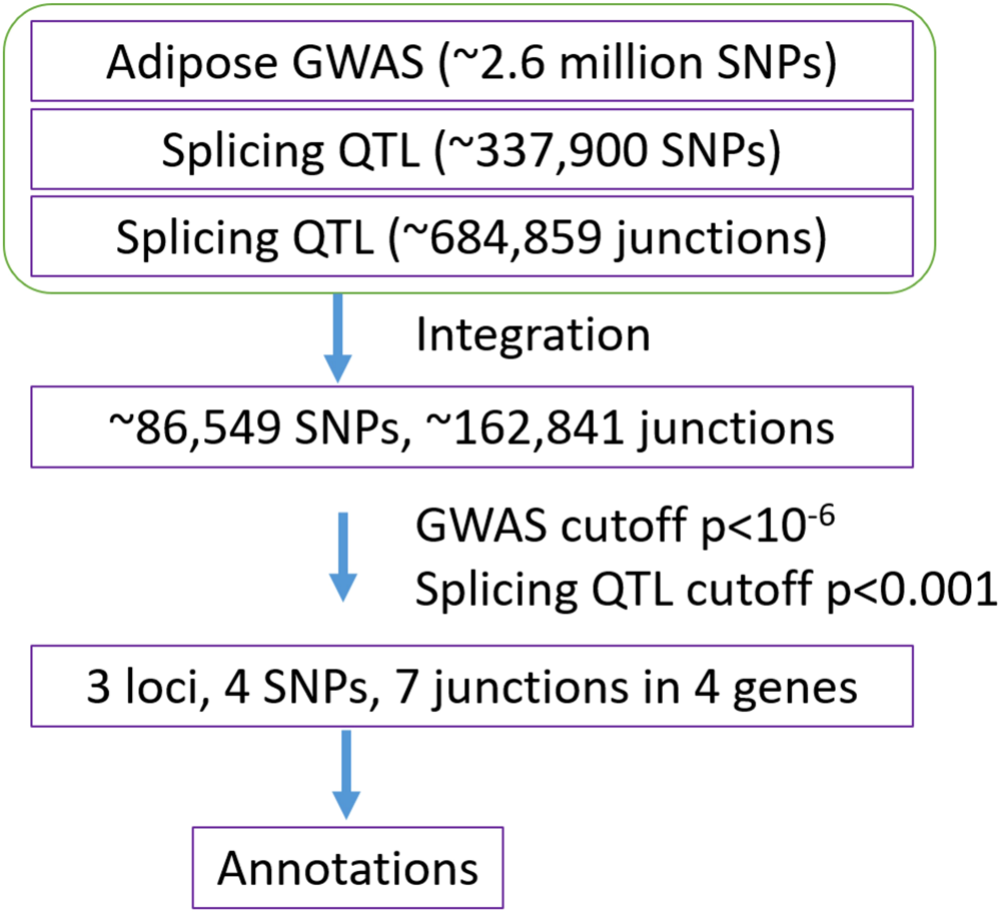
Flow chart for splicing junction identification. Note: ~, the numbers of SNPs or junctions were based on the average from multiple datasets.

Twenty-seven GWAS summary statistics datasets for 9 adipose traits with an average of 2.6 million (standard deviation (SD) = 0.2 million; range: 2.4–2.9 million) SNPs for each trait were used in this study. These nine traits are subcutaneous adipose tissue volume (SAT), visceral adipose tissue volume (VAT), visceral adipose tissue volume adjusted for BMI (VATadjBMI), pericardial adipose tissue volume (PAT), pericardial adipose tissue volume adjusted for height and weight (PATadjHtWt), subcutaneous adipose tissue attenuation (SATHU), visceral adipose tissue attenuation (VATHU), ratio of visceral-to-subcutaneous adipose tissue volume (VATSAT), and ratio of visceral-to-subcutaneous adipose tissue volume adjusted for BMI (VATSATadjBMI). For each trait, GWAS was conducted for all samples and for male and female samples respectively, resulting in 3 sets of GWAS summary statistics. Splicing QTL for 10 tissues from GTEx Pilot V3 were downloaded. We used data for six tissues that are related to adipose traits (see Methods). Specifically, 337,900 (SD = 78,241; range: 216,059–414,058) unique SNPs for each tissue from the GTEx Pilot V3 release were included, involving 684,859 (SD = 157,957; range 448,085–830,970) exon-exon links (junctions). By integrating the two datasets, we identified 86,549 (SD = 20,146; range: 51,882–111,640) overlapped SNPs. The overlapped SNPs are involved in 162,841 (SD = 37,547; range: 100,259–210,213) junctions for each tissue. The work flow is summarized in Fig. 1.

### Splicing junctions in overall samples

**Figure 2:**
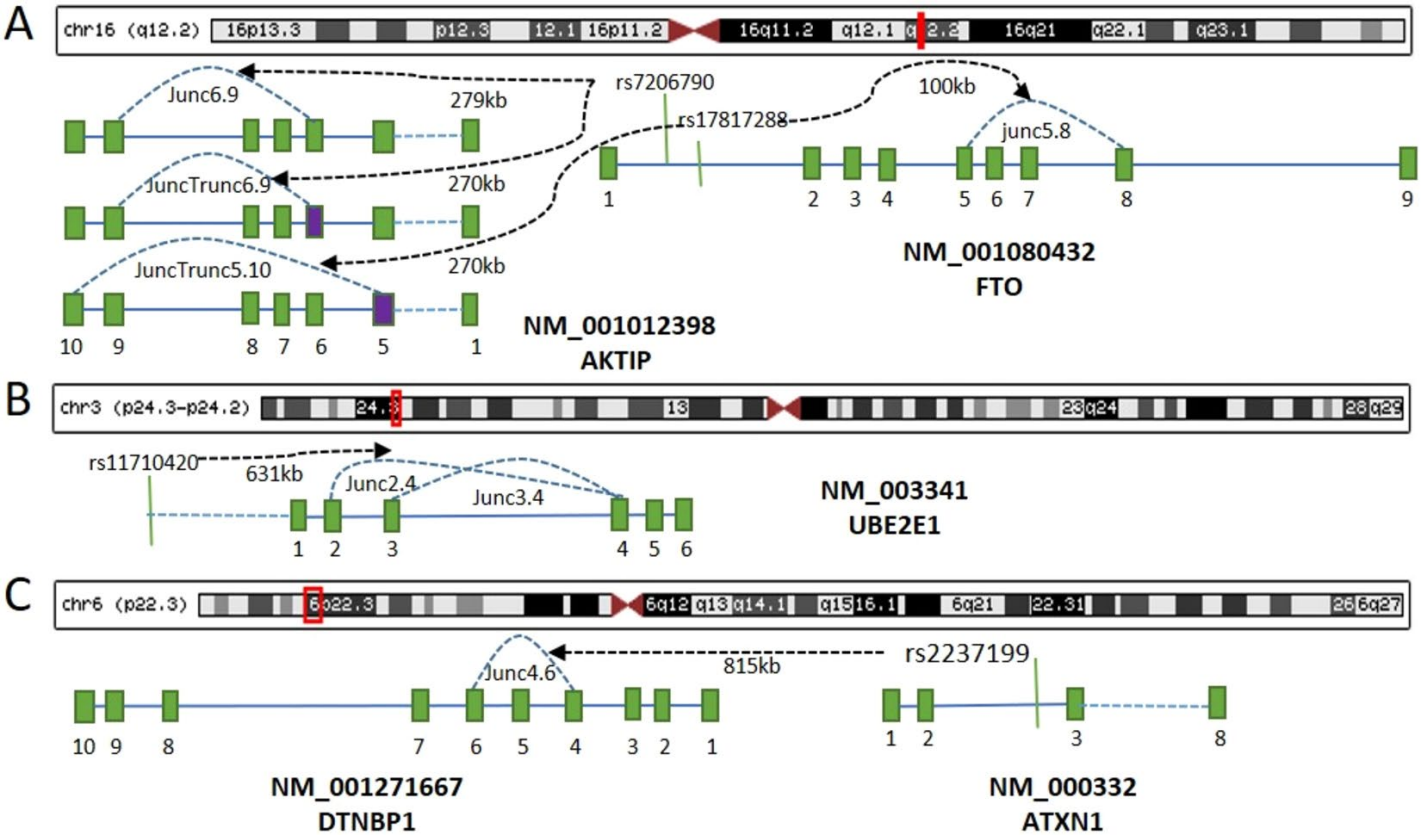
Schematic view of the 7 junctions in 3 adipose GWAS loci. (**A**) rs17817288 was associated with junc5.8 of *FTO* in the combo of SAT_OVERALL GWAS Adipose Subcutaneous, and juncTrunc5.10 of *AKTIP* in the combo of SAT_OVERALL GWAS and Artery Tibial. SNP rs7206790 was associated with junc6.9 and juncTrunc6.9 of *AKTIP* in the combo of SAT_OVERALL GWAS and Thyroid. (**B**) SNP rs11710420 was associated with junc2.4 and junc3.4 of *UBE2E1* in the combo of VATSAT_OVERALL GWAS and Whole Blood. (**C**) SNP rs2237199 was associated with junc4.6 of *DTNBP1* in the combo of SATHU_MEN GWAS and Artery Tibial. Rec box: genomic locus in chromosome; green box: exon; purple box: truncated exon. Solid green line: SNP location; solid blue line: intron; and dash line: multiple exons and introns. Black arrow, distance from SNP to junction. Junc: exon-exon junction; juncTrunc: truncated exon-exon junction.

To identify the functional body fat distribution related junctions, summary statistics of nine adipose traits GWAS and 6 GTEx tissues’ splicing QTLs were integrated. At the genome-wide significant level, which is p-value < 5.78 × 10^−7^ considering 86,549 SNPs, we did not find any significant splicing variants. Considering that this is a stringent multiple test correction, we chose a less stringent threshold to explore the splicing junctions. Specifically, we chose GWAS SNPs with p < 10^–6^ and splicing QTL with p < 0.001 for exploring the potential causal junctions that are related to adipose GWAS signals. As shown in Fig. 2 and Supplementary Table S2, for overall samples (men and women), we found two SAT GWAS SNPs (rs17817288 [p(GWAS) = 9.50 × 10^−9^] and rs7206790 [p(GWAS) = 4.20 × 10^−9^]) and one VATSAT GWAS SNP (rs11710420 [p(GWAS) = 3.20 × 10^−8^]) were significantly associated with specific expressed junctions in adipose-related tissues. (1) SNP rs17817288 was significantly associated with splicing events of exon 5 - exon 8 junction of *FTO* gene in adipose subcutaneous (p(splicing QTL) = 6.92 × 10^−4^). (2) SNP rs17817288 was significantly associated with splicing events of truncated exon 5 - exon 10 junction of *AKTIP* (AKT interacting protein) gene in artery tibial (p(splicing QTL) = 5.52 × 10^−5^). (3) SNP rs7206790 was significantly associated with splicing events of truncated and normal exon 6 – exon 9 junctions of *AKTIP* gene in thyroid (p(splicing QTL) = 7.31 × 10^−4^ and p(splicing QTL) = 5.73 × 10^−4^, respectively). (4) SNP rs11710420 was significantly associated with splicing events of exon 2 – exon 4 junction (p(splicing QTL) = 9.19 × 10^−4^) and exon 3 – exon 4 junction (p(splicing QTL) = 4.09 × 10^−4^) of *UBE2E1* gene (encoding ubiquitin conjugating enzyme E2 E1) in whole blood.

### Gender-specific splicing junctions

Gender fat pattern in total and regional body composition is apparent in adults, with men having greater lean tissue mass and a more central fat pattern compared with the more peripheral fat distribution typically observed in adult women^11^. To identify the gender-specific splicing events that are associated with the GWAS loci for body fat distribution, we used the same pipeline as the overall samples for the 9 traits. We found that SNP rs2237199, a male-specific GWAS signal that was associated with SATHU (p(GWAS) = 1.40 × 10^–8^), was significantly associated with the splicing event of exon 4 – exon 6 junction of *DTNBP1* gene (encoding dystrobrevin binding protein 1) (p = 8.30 × 10^–4^) (Fig. 2 and Supplementary Table S2). The splicing results of the 7 junctions across the 27 GWAS summary statistics datasets for body fat distribution traits were shown in Supplementary Figure S1 and Supplementary Table S3.

### Linkage disequilibrium (LD) of the splicing QTL and top SNPs in GWAS

Considering that three of the four identified GWAS SNPs were missing in the GTEx Pilot V3 splicing QTL, LD was estimated using European population by LDlink (https://analysistools.nci.nih.gov/LDlink/?tab=home). We found the LDs between the identified splicing QTLs and the 11 GWAS top SNPs were strong. The LD values of splicing QTLs (rs17817288 and rs7206790) and GWAS genome-wide significant SNP rs7185735 (chr16:53822651, hg19) were: D′ = 0.937 and 1; *r* ^2^ = 0.604 and 0.628, respectively. Moreover, the LD values of the splicing QTLs (rs11710420) and GWAS top signal rs7374732 (chr3:23203454, hg19) were: D′ = 0.937 and *r* ^2^ = 0.661. Our examination found one of the four identified SNP, rs2237199, was among the 11 GWAS top hits (Supplementary Tables S1 and S2).

## Discussion

Recent genome-wide association studies have identified 11 loci (i.e. SNPs) associated with fat tissue distributions. However, until most recently, little has known about the molecular function for most of the genome-wide significant SNPs with the phenotype in examination. In this study, by integrating GWAS summary statistics of nine body fat distribution traits with splicing QTL data from six adipose metabolism related tissues, we detected that 7 splicing events in four genes (*AKTIP*, *DTNBP1, FTO*, and *UBE2E1*) were significantly associated with four GWAS top signals for body fat distribution (rs17817288, rs7206790, rs11710420 and rs2237199; all were genome-wide significant). Our finding, although needs further extensive validation, promoted our understanding of the underlying regulatory mechanism of body fat distribution.

Fat tissue distribution has been reported to be strongly related to the development of type 2 diabetes^1^, coronary artery disease^2^, blood, and serum lipids^12^. The more recent hypothesis-free GWAS of those traits have systematically evaluated the predisposed genomic loci by using more than 10,000 human subjects (Supplementary Table S4). For adipose tissue distribution assessment to be clinically useful, the ideal adiposity phenotype should provide a single risk estimate that captures the separate ‘effects’ of diverse adiposity traits. The currently available largest studies have the greatest statistical power, and allow direct comparison to other related traits with available GWAS data (cross-trait analysis). Supplementary Table S4 showed that rs7206790 and rs17817288 reached genome-wide significant level for Type 2 diabetes among other 6 adipose-related traits, which indicated that the two SNPs shared genetic contributions to both adipose tissue distribution and type 2 diabetes (i.e. pleiotropy), while rs11710420 is likely a unique marker for adipose tissue distribution. The genetic heritability should be considered as a variable for differentiated splicing regulations.

In addition to body fat distribution traits, rs17817288 was also reported to be associated with predisposition to obesity in multiple populations^13–15^. And the associations with the combined samples were mostly contributed by the Caucasian group due to great genetic differentiation^16^. And this SNP was also reported to be associated with cancer^17^. SNP rs7206790 was reported to be associated with risk of obesity^18^ and breast cancer risk^19^. No other traits were reported with their susceptibilities from SNPs rs11710420 and rs2237199.

In this study, 6 adipose-related tissues (adipose subcutaneous, artery tibial, blood, heart left ventricle, muscle-skeletal and thyroid) were selected for splicing QTL investigation that are related to body fat distribution GWAS signals. Obesity is one of the most pervasive, chronic diseases, which is defined as excess adipose tissue. It is a significant independent risk factor for cardiovascular disease and other co-morbidities^20^. Adipose tissue is considered the best indicator of long-term essential fatty acid intake, but other tissues may prove equally valid such as whole blood^21^. Ectopic-fat depots are associated with cardio metabolic risk and cardiovascular events^22^. Pericardial adipose tissue represents distinct ectopic-fat deposition around the heart. The maintenance of energy balance is regulated by complex homeostatic mechanisms, including those emanating from adipose tissue. The main function of the adipose tissue is to store the excess of metabolic energy in the form of fat. Thyroid hormone regulates metabolic processes essential for regulating metabolism in the adult^23^. Thyroid dysfunction has a great impact on lipids as well as a number of other cardiovascular risk factors.

In this study, splicing events within the four genes were identified to be strongly associated with adipose GWAS. *FTO* (fat mass- and obesity-associated) is the most investigated gene in obesity and has complex molecular mechanisms that are yet to be elucidated. The genetic variation in the *FTO* gene was associated with metabolisms traits such as increased body weight, total fat mass, lean body mass, increased insulin secretion, and reduced insulin sensitivity^24^. In mouse models, inactivation of the *FTO* gene results in lean phenotype, whereas overexpression of *FTO* leads to increased food intake and obesity. The function of genes *AKTIP* and *UBE2E1* (encoding ubiquitin conjugating enzyme E2 E1) is still unclear. *DTNBP1* is one of most studied genes that contributes to the risk of mental health disorders. It was speculated to be involved in the modulation of glutamatergic neurotransmission in the brain^25^.

Traditional expression QTLs mainly focused on gene-level, but each gene can produce a multitude of splicing variants or isoforms. Furthermore, different isoform may be expressed in different tissues or at different developmental periods and, thus, exert unique biological functions. The accumulation of RNA-seq data especially in different tissues and disease conditions will further consolidate the tissue-specificity of some of these junctions and their corresponding isoforms. We identified 7 splicing junctions that represented a specific cluster of transcripts for the genes, which might be the underlying effect factors, considering that each gene typically has multiple transcripts. Interestingly, no significant eQTLs were found for the four SNPs in all tissues based on GTEx Portal (https://www.gtexportal.org/home/), suggesting the subtle and specific regulation of potential genetic isoforms in the adipose developmental process. There are a few limitations in the current study. First, LD trimming was not performed when examining overlapped SNPs between GWAS and sQTLs. The LD values were estimated when splicing SNPs were identified to be related to adipose-associated variations. The 7 identified junctions could not survive after multiple test corrections if we applied stringent Bonferroni correction method for the total of 162,841 junctions (Bonferroni or false discovery rate corrected significant cutoff p-value = 3.07 × 10^–7^). The experimental validation for the isoforms is also needed considering the complexity of the traits. For example, nominal significance was found for *NRG3* isoforms^26^ and *NRG1* isoforms^27^. And replications should be performed when other adipose RNA-seq samples are available. Importantly, the junctions we identified here are based on low SNP density, and until fine-mapping and functional investigations are completed it remains unclear whether their association with body fat distribution is driven by other causal variants in the region. Furthermore, current splicing QTLs provide an incomplete picture of splicing variants involved in body fat distribution, and additional adipose genetic transcript-specific junctions will be discovered as sample size increases.

To the best of our knowledge, this is the first systematical investigation of splicing architecture using genome-wide loci associated with the body fat distribution. We refined and identified 7 splicing junctions in *AKTIP*, *DTNBP1, FTO*, and *UBE2E1*, thereby providing a window into the biological processes that cause the fat tissue distribution traits. Future studies are necessary to validate these 7 junctions, and the full-length transcript clusters that cover those junctions should be determined and further explored for their mechanisms on how these transcripts affect adipocyte biology and how their perturbations contribute to systemic metabolic disease.

## Methods

### Splicing QTL datasets

Splicing QTL datasets were downloaded from GTEx Portal in section GTEx Analysis Pilot V3 (https://www.gtexportal.org/home/datasets)^10^. GTEx provides splicing QTLs for 10 tissues. In our study, we chose six GTEx tissues that were likely related to adipose-related phenotypes: adipose subcutaneous (n = 94), artery tibial (n = 112), blood (n = 156), heart left ventricle (n = 83), muscle-skeletal (n = 138), and thyroid (n = 105). The genotyping data for these tissue samples were generated by Illumina’s Human Omni5-Quad and InfiniumExomeChip. After imputation, a total of 6,820,471 autosomal SNPs were used for splicing QTL analysis. Splicing QTLs were calculated for each of the 6 tissues that had sufficient sample size (>80 donors) for all SNPs within ±1 Mb of the transcription start site of each gene^10^.

### GWAS datasets

GWAS summary statistics data from meta-analyses were downloaded from the VATGen Consortium^6^ for the following traits: SAT, VAT, VATadjBMI, PAT, PATadjHtWt, SATHU, VATHU, VATSAT, and VATSATadjBMI. The original genotyping data were generated on multiple platforms and imputation was performed based on the HapMap Phase 2 data, resulting in ~2.6 million SNPs.

### Integration and annotation

All data analyses were performed using R software with version 3.4.1. The SNP and gene coordinates were based on human reference genome hg19, and reference transcripts were referred to UCSC Genome Browser and NCBI RefSeqGene (*Homo sapiens* Release 108, 2016-06-07).

## Electronic supplementary material

Supplementary file
