# Supplementary material for: Splicing QTL of human adipose-related traits
Source: Sci Rep. 2018 Jan 10;8:318. doi: 10.1038/s41598-017-18767-z (PMC5762880; doi:10.1038/s41598-017-18767-z)
Supplement: Supplementary file 1 — Supplementary file [file 41598_2017_18767_MOESM1_ESM.pdf]

Supplementary file

Splicing QTL of human adipose-related traits

Liang Ma[1], Peilin Jia[1], Zhongming Zhao[1,2,*]

[1]Center for Precision Health, School of Biomedical Informatics, The University of Texas Health Science Center at Houston, Houston, TX, USA

[2]Human Genetics Center, School of Public Health, The University of Texas Health Science Center at Houston, Houston, TX, USA

Supplementary Table S1. HaploReg information of 11 top SNPs of adipose GWAS (PMID: 27918534)

Chr.	SNP ID	Pos. (bp, hg38)	Pos. (bp, hg19)	Ref. allele	Alt. allele	Minor allele frequency				RefSeq gene
						AFR	AMR	ASN	EUR	
chr1	rs6587515	150636412	150608888	G	A	0	0.04	0.18	0.08	6.8kb 5' of *ENSA*
chr1	rs6689335	219455340	219628682	T	C	0.03	0.32	0.30	0.49	242kb 3' of *LYPLAL1*
chr2	rs10198628	12824371	12964497	A	G	0.6	0.51	0.84	0.55	82kb 3' of *TRIB2*
chr3	rs7374732	23161963	23203454	C	T	0.93	0.72	0.65	0.65	41kb 5' of *UBE2E2*
chr5	rs10060123	126348218	125683910	C	A	0.12	0.20	0.20	0.26	12kb 5' of *GRAMD3*
chr5	rs1650505	158602726	158029734	G	A	0.22	0.28	0.24	0.20	93kb 3' of *EBF1*
chr6	rs912056	6735964	6736197	A	T	0.82	0.56	0.25	0.64	81kb 3' of *LY86*
chr6	rs2842895	7106083	7106316	G	C	0.04	0.46	0	0.56	1.5kb 5' of *RREB1*
chr6	rs2237199	16429790	16430021	G	A	0.18	0.17	0.37	0.12	*ATXN1*
chr16	rs7185735	53788739	53822651	A	G	0.54	0.28	0.16	0.42	*FTO*
chr17	rs2123685	39897636	38053889	T	C	0.01	0.03	0	0.04	7kb 3' of *GSDMB*

AFR: African. AMR: Ad Mixed American. ASN: Asians. EUR: European.

Supplementary Table S2. Splicing QTL for adipose traits

SNP ID	Position (bp)	GWAS_pval	Splicing feature									Distance (bp)
			Trait-Tissue	Gene	Exon x1	Exon x2	Junction Coordinate	Direction	Junction	sQTL_pval	RefGene ID	
rs17817288	53807764	9.50E-09	SAT_OVERALL-AdiposeSubcutaneous	FTO	Exon 5/9	Exon 8/9	53907777-53967942	fwd	Novel	6.92E-04	NM_001080432	100,013
rs17817288	53807764	9.50E-09	SAT_OVERALL-ArteryTibial	AKTIP	Truncated Exon 5/10	Exon 10/10	53526431-53529017	fwd	Novel	5.52E-05	NM_001012398	281,333
rs7206790	53797908	4.20E-09	SAT_OVERALL-Thyroid	AKTIP	Exon 9/10	Truncated Exon 6/10	53526665-53528483	rev	Novel	7.31E-04	NM_001012398	271,243
rs7206790	53797908	4.20E-09	SAT_OVERALL-Thyroid	AKTIP	Exon 9/10	Exon 6/10	53526665-53528429	rev	Novel	5.73E-05	NM_001012398	271,243
rs11710420	23221931	3.20E-08	VATSAT_OVERALL-WholeBlood	UBE2E1	Exon 3/6	Exon 4/6	23853001-23929058	rev	Known	9.19E-04	NM_003341	631,070
rs11710420	23221931	3.20E-08	VATSAT_OVERALL-WholeBlood	UBE2E1	Exon 2/6	Exon 4/6	23848912-23929058	rev	Known	4.09E-04	NM_003341	626,981
rs2237199	16430271	1.40E-08	SATHU_MEN-ArteryTibial	DTNBP1	Exon4/10	Exon6/10	15615630-15637975	fwd	Novel	8.30E-04	NM_001271667	814,641

SAT, subcutaneous adipose tissue volume; VATSAT, ratio of visceral-to-subcutaneous adipose tissue volume; SATHU, Subcutaneous adipose tissue attenuation.

Distance: base pair from SNP to junction.

Supplementary Table S3. Four splicing QTL GWAS p-values for body fat distribution traits

Trait	Adipose GWAS p-value			
	rs17817288	rs7206790	rs11710420	rs2237199
PAT_MEN	7.80E-04	0.007	0.001	0.072
PAT_OVERALL	0.001	0.011	0.160	0.590
PAT_WOMEN	0.480	0.530	0.120	0.640
PATadjHtWt_MEN	0.046	0.160	9.60E-04	0.053
PATadjHtWt_OVERALL	0.130	0.150	0.036	0.920
PATadjHtWt_WOMEN	0.610	0.870	0.540	0.420
SAT_MEN	2.50E-06	1.40E-06	0.370	1.80E-04
SAT_OVERALL	9.50E-09	4.20E-09	0.650	0.008
SAT_WOMEN	5.20E-04	3.40E-04	0.780	0.820
SATHU_MEN	0.079	0.006	0.500	1.40E-08
SATHU_OVERALL	0.016	4.00E-04	0.069	0.002
SATHU_WOMEN	0.094	0.018	0.062	0.360
VAT_MEN	8.10E-05	0.002	0.400	0.001
VAT_OVERALL	7.60E-05	0.001	0.001	0.004
VAT_WOMEN	0.069	0.130	9.70E-05	0.480
VATadjBMI_MEN	0.680	0.820	0.110	0.036
VATadjBMI_OVERALL	0.990	0.740	6.10E-04	0.012
VATadjBMI_WOMEN	0.540	0.690	0.003	0.110
VATHU_MEN	0.210	0.100	0.840	8.00E-06
VATHU_OVERALL	0.036	0.007	0.170	0.009
VATHU_WOMEN	0.086	0.026	0.067	0.680
VATSAT_MEN	0.300	0.150	0.014	0.930
VATSAT_OVERALL	0.140	0.019	3.20E-08	0.370
VATSAT_WOMEN	0.310	0.055	2.60E-07	0.320
VATSATadjBMI_MEN	0.390	0.200	0.022	0.770
VATSATadjBMI_OVERALL	0.120	0.010	3.10E-07	0.290
VATSATadjBMI_WOMEN	0.210	0.027	2.60E-06	0.340

Supplementary Table S4. GWAS p-values of the four splicing QTLs for the adipose-related traits

PMID	Trait	GWAS p-value				Sample size	Ancestry
		rs17817288	rs7206790	rs11710420	rs2237199		
27918534	Adipose	9.50E-09	4.20E-09	3.20E-08	1.40E-08	18,332 (9,594 women/8,738 men)	European, African, Hispanic, and Chinese
27398621	Type 2 diabetes	2.88E-10	9.22E-12	0.7031	NA	12,940 (11,645 T2D cases/32,769 controls)	European, African, Hispanic, and Asian
26343387	Coronary artery disease	0.0232	0.0667	0.4568	0.4289	184,305 (60,801 cases/123,504 controls)	European, African Americans, Hispanic, South Asian, and East Asian
24097068	HDL	0.3028	3.92E-06	2.80E-05	0.9663	188,577 Europeans/7,898 non-Europeans	European, African, East Asian, and South Asian
	LDL	0.7714	0.9233	0.4601	0.6172		
	Total cholesterol	0.7378	0.4435	0.2637	0.6275		
	Triglycerides	0.3297	7.98E-05	4.05E-04	0.9626		

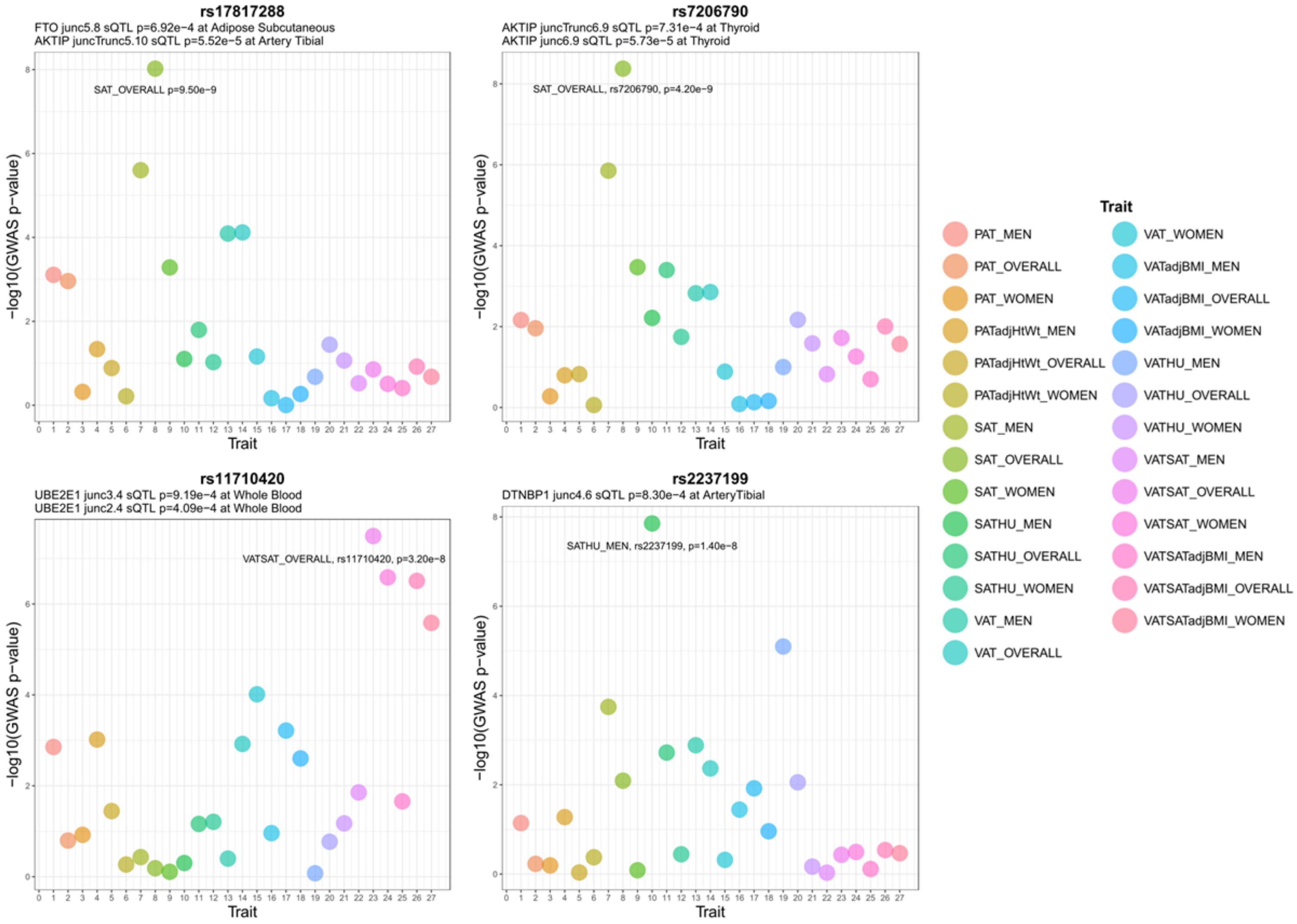

Supplementary Figure S1. The p-value distributions of 4 splicing junction SNPs in 27 GWAS including 9 traits in 3 groups (male, female and overall). Note: each dot represents a combo of trait and group. SAT, subcutaneous adipose tissue volume; VAT, visceral adipose tissue volume; VATadjBMI, visceral adipose tissue volume adjusted for BMI; PAT, pericardial adipose tissue volume; PATadjHtWt, pericardial adipose tissue volume adjusted for height and weight; SATHU, subcutaneous adipose tissue attenuation; VATHU, visceral adipose tissue attenuation; VATSAT, ratio of visceral-to-subcutaneous adipose tissue volume; and VATSATadjBMI, ratio of visceral-to-subcutaneous adipose tissue volume adjusted for BMI.
